# HER3-targeted therapy: the mechanism of drug resistance and the development of anticancer drugs

**DOI:** 10.20517/cdr.2024.11

**Published:** 2024-04-29

**Authors:** Huilan Zeng, Wei Wang, Lin Zhang, Zhenghong Lin

**Affiliations:** ^1^School of Life Sciences, Chongqing University, Chongqing 401331, China.; ^2^Department of Cancer Center, Chongqing University Three Gorges Hospital, School of Medicine, Chongqing University, Chongqing 404000, China.; ^3^Department of Gastroenterology, Chongqing University Jiangjin Hospital, Chongqing 402260, China.

**Keywords:** HER3, molecular mechanism, drug resistance, targeted therapy, monoclonal antibody, molecular target

## Abstract

Human epidermal growth factor receptor 3 (HER3), which is part of the HER family, is aberrantly expressed in various human cancers. Since HER3 only has weak tyrosine kinase activity, when HER3 ligand neuregulin 1 (NRG1) or neuregulin 2 (NRG2) appears, activated HER3 contributes to cancer development and drug resistance by forming heterodimers with other receptors, mainly including epidermal growth factor receptor (EGFR) and human epidermal growth factor receptor 2 (HER2). Inhibition of HER3 and its downstream signaling, including PI3K/AKT, MEK/MAPK, JAK/STAT, and Src kinase, is believed to be necessary to conquer drug resistance and improve treatment efficiency. Until now, despite multiple anti-HER3 antibodies undergoing preclinical and clinical studies, none of the HER3-targeted therapies are licensed for utilization in clinical cancer treatment because of their safety and efficacy. Therefore, the development of HER3-targeted drugs possessing safety, tolerability, and sensitivity is crucial for clinical cancer treatment. This review summarizes the progress of the mechanism of HER3 in drug resistance, the HER3-targeted therapies that are conducted in preclinical and clinical trials, and some emerging molecules that could be used as future designed drugs for HER3, aiming to provide insights for future research and development of anticancer drugs targeting HER3.

## INTRODUCTION

Cancer ranks as the primary cause of mortality and a significant impediment to raising life expectancy worldwide^[1]^. Due to the frequent emergence of drug resistance after several cycles of chemotherapy, cancer treatment often becomes inefficient^[2]^. Therefore, discovering new targets and developing new drugs are essential for cancer therapy.

Human epidermal growth factor receptor 3 (HER3/ErbB3) is a tyrosine kinase receptor belonging to the HER/ErbB receptor tyrosine kinase (RTK) family, along with the epidermal growth factor receptor EGFR/HER1, HER2/ErbB2/neu and HER4/ErbB4 in mammals^[3,4]^. Structurally, the HER3 protein comprises an extracellular domain (ECD) that is responsible for binding ligands, which encompasses subdomains I-IV. HER3 also contains a transmembrane segment with hydrophobic properties and an intracellular domain housing a juxtamembrane region, a segment with tyrosine kinase activity, and a carboxyterminal tail rich in tyrosine residues^[5,6]^. Neuregulins (NRGs) 1-2, also known as heregulins (HRGs), are the preferred ligands for HER3^[7]^. In the absence of a ligand, HER3 adopts an inactive conformation as a monomer. However, when a ligand attaches to subdomains I and III, HER3 undergoes a structural shift and exposes its arm for dimerization, allowing it to interact with another monomer and form a heterodimer^[3,8]^. HER3 prefers to dimerize with HER family members, including EGFR and HER2, but with a weak affinity to HER4^[8]^. Additionally, HER3 can dimerize with non-HER receptors, such as mesenchymal-epithelial transition (MET) factor receptor, fibroblast growth factor receptor 2 (FGFR2), and insulin-like growth factor receptor 1 (IGF-1R)^[9-11]^. HER3 is hard to create a homodimer and only possesses weak intracellular tyrosine kinase activity since it differs at crucial residues within the kinase domain, resulting in its confinement in an inactive-like conformation^[12,13]^. Upon ligand binding, the kinase domain of the dimerization partner phosphorylates the tyrosine residues in the C-terminal tail of HER3, subsequently initiating downstream signaling cascades^[14]^.

**Figure 1:**
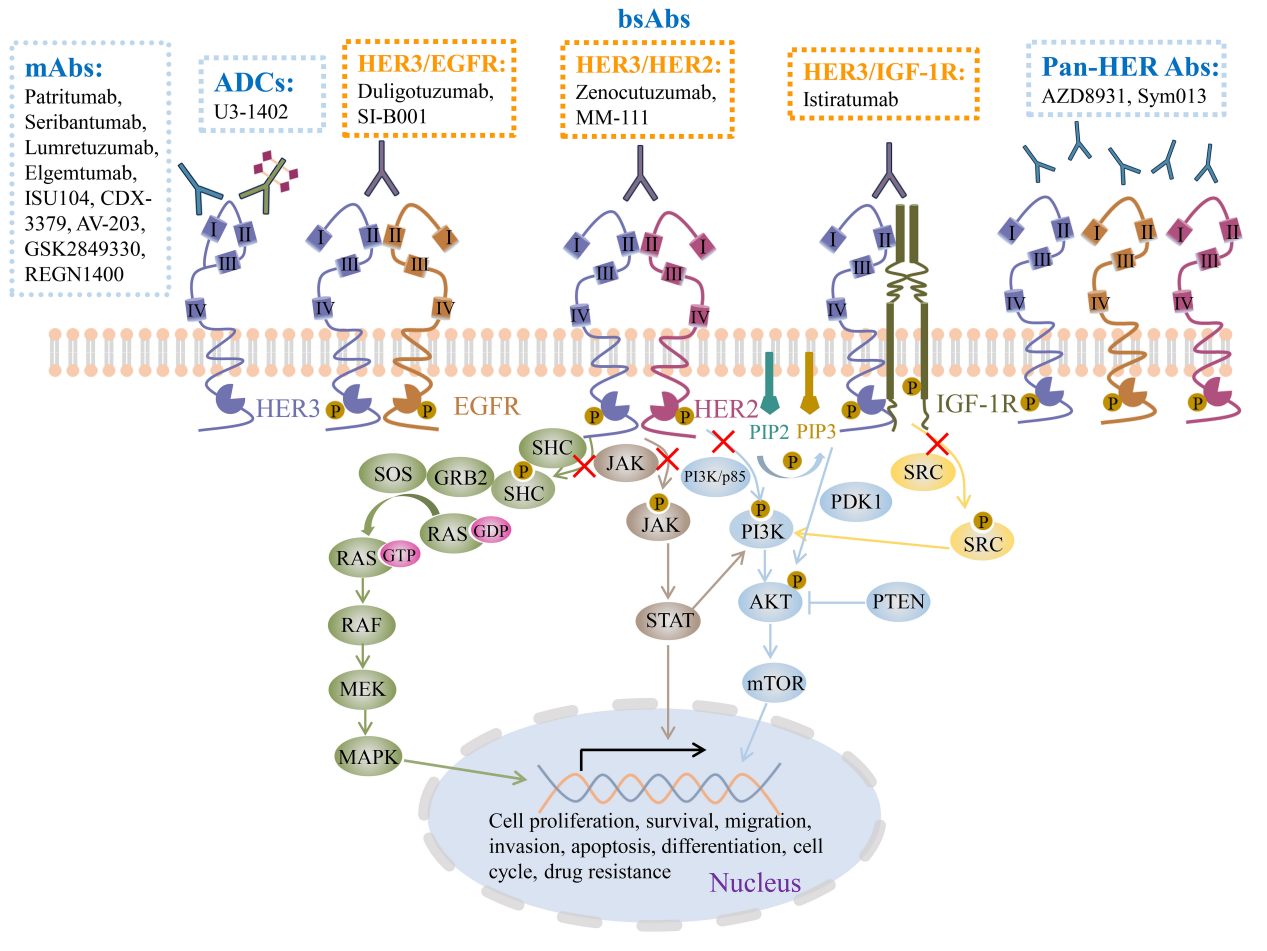
HER3 targeted therapies in the clinic and HER3 downstream signaling pathways. At present, the treatment strategies targeting HER3 in clinical trials mainly include monoclonal antibodies, bispecific antibodies, and antibody-drug conjugates. Among them, monoclonal antibodies are the first developed agents with the most types. Since HER3 only has weak intracellular tyrosine kinase activity, HER3 is hard to be autophosphorylated but forms dimers with other receptors, including EGFR, HER2, and IGF-1R, especially HER2, and is phosphorylated by dimerization partner through transphosphorylation. HER2/HER3 dimers can activate PI3K/AKT, JAK/STAT, and MEK/MAPK pathways, but the Src kinase pathway is predominantly mediated by the IGF-1R/HER3 dimers. In addition, Src kinase and STAT protein can upregulate PI3K expression levels. Downstream effectors in the pathways such as mTOR, STAT, and MAPK can translocate into the nucleus and control the expression of multiple genes implicated in various processes involved in cancer development, such as cancer cell proliferation, survival, migration, invasion, apoptosis, differentiation, angiogenesis, cell cycle, and drug resistance. However, when antibodies bind to HER3 monomer or heterodimer, the signaling pathway downstream of HER3 is blocked, subsequently inhibiting the cancer progression. mAbs: Monoclonal antibodies; ADCs: antibody-drug conjugates; HER: human epidermal growth factor receptor; EGFR: epidermal growth factor receptor; IGFR: insulin-like growth factor receptor 1; MAPK: mitogen-activated protein kinase; MEK: mitogen-activated extracellular signal-regulated kinase; RAF: rapidly accelerated fibrosarcoma; RAS: rat sarcoma; GTP: guanosine triphosphate; SOS: son of sevenless; GRB2: growth factor receptor-bound protein 2; P: phosphorylation; SHC: src homolog and collagen homolog; JAK: janus tyrosine kinase; STAT: signal transducers and activators of transcription; PI3K: phosphatidylinositide 3-kinases; AKT: also named PKB (protein kinase B); mTOR: mammalian target of rapamycin; PIP2: phosphatidylinositol-4,5-bisphosphate; PIP3: phosphatidylinositol-3,4,5-trisphosphate; PDK1: 3-phosphoinositide-dependent protein kinase-1; SRC: proto-oncogene tyrosine-protein kinase SRC; PTEN: phosphatase and tensin homolog.

HER3 plays a prominent role in the field of cancer biology [Figure 1]. HER3 expression is linked to cell proliferation, invasion, metastasis, and poor overall survival in various cancer types, including breast^[15]^, prostate^[16]^, lung^[17]^, colorectal^[18]^, melanoma^[19]^, ovarian^[20]^, gastric^[21]^, pancreatic^[22]^, head and neck cancer^[23]^. Moreover, HER3 can collaborate with other HER receptors to activate downstream signaling, such as activation of the PI3K/AKT, JAK/STAT, and MEK/MAPK pathways by HER2/HER3, or activation of the Src pathway by IGF-IR/HER3. The downstream effectors enter the nucleus to regulate the expression of relevant genes, which ultimately leads to a wide range of processes, such as drug resistance^[24]^. Overall, HER3 can be a highly promising target for cancer treatment.

Herein, we summarized the progress of the mechanisms of HER3 in drug resistance, current therapeutic approaches, and some potential molecules targeting HER3, aiming to provide new insights into drug design and cancer therapy for HER3.

## THE MECHANISM OF HER3 IN DRUG RESISTANCE

**Figure 2:**
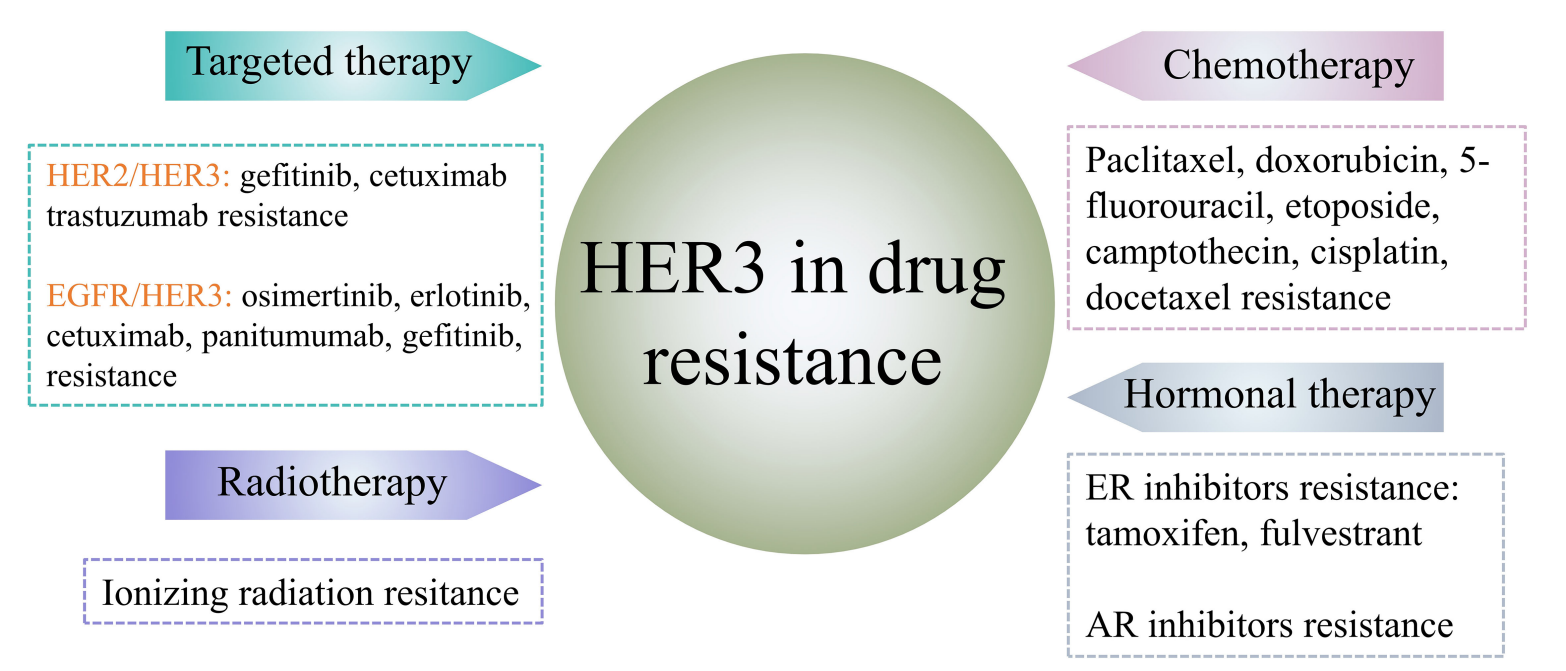
HER3 is involved in drug resistance in the treatment processes of different cancers. The dimerization of HER3 with HER2 or EGFR is involved in the resistance of multiple drugs in cancer-targeted therapy. In addition, HER3 also participates in multidrug resistance in cancer chemotherapy, hormonal therapy, and radiotherapy resistance. HER: Human epidermal growth factor receptor; ER: estrogen receptor; AR: androgen receptor; EGFR: epidermal growth factor receptor.

Since HER3 has six phosphotyrosine sites on its intracellular C-terminal tail, it is more likely to be phosphorated. Phosphorylated HER3 can bind to the p85 subunit (SH2 domain) of PI3K and then activate PI3K/AKT signaling^[14]^. PI3K/AKT signaling is a critical pathway for cell survival and has a strong association with multidrug resistance in many cancers^[25]^. Except for PI3K/AKT signaling, MEK/MAPK signaling is also a principal downstream signaling pathway of HER3^[26]^. Mitogen-activated protein kinase (MAPK) regulates almost all aspects of physiological processes and is frequently altered in disease. In cancer, MAPK can control cell proliferation, differentiation, survival, migration, invasion, senescence, apoptosis, inflammation, and drug resistance^[27,28]^. Therefore, HER3 plays a critical role in drug resistance [Figure 2].

### The mechanism of HER3 in resistance to targeted therapy

Because HER3 has a strong tendency to form heterodimers with HER2, ErbB2/ErbB3 heterodimer can promote HNSCC cell growth and increase resistance to EGFR TKI gefitinib^[29]^. Heterotrimerization of erbB3/erbB2/IGF-IR blocked trastuzumab binding and activated PI3K/AKT signaling pathway and Src kinase, which resulted in trastuzumab resistance in breast cancer cells^[30]^. In addition, stimulation with NRG1 activated PI3K/AKT and MEK/MAPK pathways, which led to intrinsic resistance to trastuzumab in HER2+ BC models^[31]^. ErbB2 overexpression or high levels of NRG increases the formation of HER2/HER3 dimers, resulting in either intrinsic or acquired resistance to cetuximab-based treatment in CRC patients^[32]^.

HER3 dimer formation with EGFR is also involved in resistance to targeted therapies in several malignancies. An elevation in EGFR/HER3 dimerization was detected in BC patients, which led to PI3K/AKT signal transduction and cetuximab/panitumumab resistance^[33]^. EGFR/HER3 was activated in gefitinib-resistant CRC cells, miR-323a-3p reversed ErbB3/EGFR signaling activation and blocked acquired gefitinib resistance^[34]^. MET amplification drove HER3-dependent activation of PI3K and subsequently caused EGFR TKI gefitinib and erlotinib resistance in NSCLC^[35]^.

### The mechanism of HER3 in resistance to chemotherapy

In addition to its role in resistance to targeted therapies, HER3 can also contribute to resistance to chemotherapies. In HER2+ BC, increased HER3 caused resistance to paclitaxel through survivin amplification^[36]^. Concurrent expression of HER2 and HER3 activated PI3K/AKT signaling and was connected to resistance to a variety of chemotherapeutics, including doxorubicin, paclitaxel, etoposide, 5-fluorouracil, and camptothecin in breast cancer cell lines^[37]^. Chemotherapeutic drug doxorubicin induced HRG amplification and activation of the HER3/PI3K/AKT signaling resulting in inhibiting ovarian cancer cell apoptosis. Additionally, cisplatin also upregulated HER3 in ovarian cancer cells^[38]^. Therefore, HER3 is strongly associated with chemotherapy resistance in ovarian cancer.

### The mechanism of HER3 in resistance to radiotherapy

HER3 expression is also associated with radiotherapy resistance. In human luminal A breast cancer cells, silencing HER3 improved the sensitivity of breast cancer cells to radiotherapy because it decreased cell proliferation and colony formation following exposure to ionizing radiation (IR). Mechanistically, silencing of HER3 promoted IR-induced DNA damage, decreased DNA repair, and increased apoptosis^[39]^.

### The mechanism of HER3 in resistance to hormonal therapy

Induction of HER family receptors was associated with tamoxifen resistance of estrogen receptor (ER)-positive (ER+) breast cancers^[40]^. In BC cells, the reduction of ErbB3 restored ErbB2-associated antiestrogen receptor tamoxifen sensitivity via enhanced apoptosis. The mechanism is that silencing HER3 decreases p-Akt levels and alters the phosphorylation status of ERα^[41]^. Furthermore, co-expression of HER2 and HER3 is more likely to develop tamoxifen resistance^[42]^. The activation of HER3 also contributed to the resistance of ER suppressor fulvestrant in breast cancer cells^[43]^. EGFR/HER3 dimers led to androgen receptor (AR) therapy resistance through stabilization of AR and upregulation of PI3K/AKT signaling in castration-resistant prostate cancer (CRPC)^[44]^. In addition, NRG1 produced by stromal cells was also demonstrated to increase antiandrogen resistance in CRPC^[45]^.

## THE DEVELOPMENT OF HER3 TARGETING CANCER THERAPY

Extensive endeavors are being made to devise strategies to combat cancer by targeting HER3 owing to its crucial role in tumor advancement and drug resistance^[4]^. Considering the weak enzymatic activity to target, anti-HER3 strategies have shifted from HER3 tyrosine kinase inhibitors (TKIs) to antibodies, especially monoclonal antibodies (mAbs)^[46]^. However, mAbs have some limitations, such as a lack of efficacy. The new approaches, such as bispecific antibodies (bsAbs) and antibody-drug conjugates (ADCs) targeting HER3, pan-HER strategies, and HER3 vaccinations, have been developed and created new hope for HER3-targeted therapy^[3]^.

### Monoclonal antibodies

Monoclonal antibodies (mAbs) can inhibit ligand binding on the HER3 extracellular domain, or block the HER3 dimerization partner’s kinase activity or its ability to dimerize with HER3, or suppress HER3 expression on the cell surface, or lock HER3 in an inactive conformation^[47]^. Most HER3-targeting mAbs are now being investigated in preclinical and clinical trials [Table 1]. So far, multiple mAbs targeting HER3 have been identified, with patritumab, seribantumab, and lumretuzumab being the most studied. Therefore, we mainly described these three mAbs, including their combination therapies in clinical trials.

**Table 1:** Summary of human monoclonal antibodies of HER3

**Compound**	**Format**	**Inhibiting or competing NRG binding**	**Research status**	**Main side effects**	**Ref.**
Patritumab (AMG-888/U3-1287)	Human IgG1	Inhibiting	Terminated after phase III trial	Diarrhea, rash and nausea	Forster *et al*.^[48]^
Seribantumab (MM-121/SAR256212)	Human IgG2	Competing	Phase II trial	Diarrhea, rash, mucosal inflammation, dermatitis acneiform, vomiting and nausea	Sequist *et al*.^[49]^ Cleary *et al*.^[50]^
Lumretuzumab (RG7116/RO5479599/GE-huMab-HER3)	Humanized IgG1	Inhibiting	Terminated after phase II trial	Diarrhea, gastrointestinal and skin toxicities	Cejalvo *et al*.^[51]^ Mirschberger *et al*.^[52]^
Elgemtumab (LJM716)	Human IgG1	Competing	Phase II trial	Diarrhea, nausea, vomiting and constipation	Jhaveri *et al*.^[53]^
ISU104	Human IgG1	Inhibiting	Phase I trial	Oral mucositis, pruritus, diarrhea	Hong *et al*.^[54]^
CDX-3379 (KTN3379)	Human IgG1	Inhibiting	Phase II trial	Diarrhea, nausea and rash	Duvvuri *et al*.^[55]^
AV-203 (CAN017)	Humanized IgG1	Inhibiting	Phase I trial	NA	Meetze *et al*.^[56]^
GSK2849330	Chimeric IgG1/IgG3	Inhibiting	Phase I trial	Diarrhea	Menke-van der Houven van Oordt *et al*.^[57]^
EV20	Humanized IgG1	Inhibiting	Preclinical	-	Sala *et al*.^[58]^
1A5-3D4	Humanized IgG1	Inhibiting	Preclinical	-	Wang *et al*.^[59]^
H4B-121	Human IgG1	Competing	Preclinical	-	Lazrek *et al*.^[60]^
IgG 95	Human IgG1	Inhibiting	Preclinical	-	Turowec *et al*.^[61]^
IgG 3-43	Human IgG1	Competing	Preclinical	-	Schmitt *et al*.^[62]^
10D1F	Humanized IgG1	Inhibiting	Preclinical	-	Thakkar *et al*.^[63]^

HER: Human epidermal growth factor receptor; NRG: neuregulin; IgG1: immunoglobulin G1; NA: not applicable; IgG3: immunoglobulin G3.

#### Patritumab (AMG-888, U3-1287)

Patritumab is a first-in-class, fully human immunoglobulin G1 (IgG1) mAb attaching to the HER3-ECD, which blocks HER3 ligand binding and triggers receptor internalization and degradation. Furthermore, it prevents HER3 activation and downstream signal transduction^[64]^. U3-1287 exhibits cellular migration, proliferation, colony formation, and growth suppression *in vitro* and *in vivo* xenograft models^[24,64]^. Patritumab, alone or combined with an anti-EGFR mAb, inhibited non-small cell lung cancer (NSCLC) xenografts growth, including wild-type and TKI-resistant EGFR models^[65]^. Combining patritumab with EGFR TKIs may be beneficial in treating HRG-overexpressing NSCLC patients with resistance to EGFR inhibitors, as it can overcome HRG-dependent EGFR inhibitor resistance^[66]^. Patritumab can restore cetuximab sensitivity in colorectal cancer cells that have developed cetuximab resistance due to HRG^[67]^. In addition, U3-1287/AMG888, in combination with radiation, can enhance the efficacy of radiotherapy as U3-1287/AMG888 could inhibit basal and radiation-induced activation of HER3, AKT, rpS6, and MAPK in NSCLC and head and neck squamous carcinoma (HNSCC)^[68]^. This mAb is undergoing phase I-III clinical trials, and the outcomes are promising^[3]^.

#### Seribantumab (MM-121, SAR256212)

Seribantumab (MM-121), a fully human IgG2 mAb, competes with the HRG ligand in order to bind with HER3, blocking HER2 and HER3 dimerization, inducing HER3 internalization and degradation, and subsequently inhibiting PI3K/AKT and MEK/MAPK signaling^[69]^. Seribantumab decreases tumor growth in colorectal^[70]^, pancreatic^[71]^, breast^[72]^, NSCLC^[49]^, ovarian^[73]^, bladder^[74]^ cancer models. Seribantumab was tested alone or combined with EGFR-inhibiting antibodies, chemotherapy drugs, or PI3K inhibitors in phase I and II studies^[49,75]^. MM-121 combined with trastuzumab inhibited HER2+ breast cancer (BC) cell proliferation and promoted apoptosis of trastuzumab-resistant cells ^[76]^. MM-121 combined with erlotinib inhibited pancreatic cancer cell proliferation^[77]^. MM-121, combined with cetuximab, exerted a more potent antitumor activity in HNSCC models^[78]^. Recently, some studies have suggested that higher NRG mRNA levels and lower HER2 levels predict clinical benefits from adding seribantumab to standard therapies^[79]^. Seribantumab is well tolerated and secure, and has entered into phase II clinical trials^[3,49]^.

#### Lumretuzumab (RG7116, RO5479599, GE-huMab-HER3)

Lumretuzumab, a humanized IgG1, targets subdomain I of the HER3-ECD with high affinity. The mAb almost completely blocks HRG binding and receptor heterodimerization and inhibits HER3 activation and downstream AKT phosphorylation at concentrations of 1 nmol/L^[52]^. Lumretuzumab can also attach to FcγRIIIa on immune cells with high affinity, leading to increased antibody-dependent cell-mediated cytotoxicity (ADCC)^[80]^. Lumretuzumab combined with cetuximab and erlotinib was secure, but the efficacy was mediocre in different cancers^[81]^. The combination of lumretuzumab, pertuzumab and paclitaxel treatment was linked to a high frequency of diarrhea, and the therapeutic window remained too narrow to guarantee continued clinical research in metastatic breast cancer^[82]^. Moreover, lumretuzumab combined with carboplatin and paclitaxel was well tolerated in NSCLC, and patients with high heregulin expression levels may benefit from this treatment^[51]^. Phase I trials showed that luminituzumab was well tolerated and showed therapeutic activity, but its efficacy in phase Ib/II trials needs to be demonstrated further^[51,83]^.

### Bispecific antibodies

Bispecific antibodies (bsAbs), which merge two distinct sites for binding antigens within one molecule, offer superior precision in targeting, unique mechanisms of action, and heightened clinical efficacy^[84]^. bsAbs are designed to connect immune cells to cancer cells in order to kill tumor cells and target RTKs^[85]^. To destroy the limitations and overcome drug resistance to mAbs, various bsAbs of HER3 are being investigated in preclinical and clinical trials^[86]^ [Table 2]. Next, we mainly focused on bispecific antibodies in clinical trials.

**Table 2:** Summary of bispecific antibodies of HER3

**Compound**	**Targeting receptors**	**Research status**	**Main side effects**	**Ref.**
Zenocutuzumab (MCLA-128, Zeno)	HER2 and HER3	Phase II trial	Diarrhea and rash	Alsina *et al*.^[87]^
MM-111	HER2 and HER3	Terminated after phase II trial	Diarrhea, dyspnoea, stomatitis, vomiting, alopecia, constipation and cough	Denlinger *et al*.^[88]^
Istiratumab (MM-141)	IGF1R and HER3	Phase II trial	Vomiting, nausea, diarrhea, abdominal pain, alopecia, dyspnea and rash	Kundranda *et al*.^[89]^
Duligotuzumab (MEHD7945A, RG7597)	EGFR and HER3	Phase II trial	Headache, rash and diarrhea, nausea, dermatitis acneiform	Lieu *et al*.^[90]^ Juric *et al*.^[91]^
SI-B001	EGFR and HER3	Phase II trial	-	Xue *et al*.^[92]^
DVD-Ig	EGFR and HER3	Preclinical	-	Gu *et al*.^[93]^
scDb hu225×3-43-Fc	EGFR and HER3	Preclinical	-	Rau *et al*.^[94]^
Dab-Fc	HER2 and HER3	Preclinical	-	Rau *et al*.^[95]^
1G5D2	HER2 and HER3	Preclinical	-	Hassani *et al*.^[96]^

HER: Human epidermal growth factor receptor; IGF1R: insulin-like growth factor 1 receptor; EGFR: epidermal growth factor receptor; DVD-Ig: dual variable domain immunoglobulin.

#### Zenocutuzumab (Zeno, MCLA-128)

Zenocutuzumab is a bispecific humanized IgG1 with two distinct Fab arms that target the domain I of HER2 and domain III of HER3^[86]^. Zenocutuzumab binds to HER2 arms and then prevents HER3 from going through the conformational change that is needed for HER3 to heterodimerize with HER2 and EGFR. This peculiar “dock (HER2 arm) and block (HER3 arm)” mechanism inhibits HER3 cytoplasmic domain phosphorylation and downstream oncogenic signaling transduction. Furthermore, the glycoengineered modification of IgG1 increases affinity for Fc receptors, leading to increased ADCC, which enables the recruitment of natural killer effector cells and subsequent elimination of tumor cells^[97]^. Zenocutuzumab was demonstrated to antagonize pancreatic, gastric, and breast cancer effectively, even for those resistant to trastuzumab and T-DM1 and with high NRG levels^[3]^. Trials for zenocutuzumab in phases I and II are now underway, where it is shown to have a tolerable toxicity profile and anticancer effectiveness^[86]^.

#### MM-111

MM-111 could target HER2 and HER3 simultaneously, resulting in the synthesis of a receptor/MM-111 complex and suppression of cell growth^[98]^. This bsAb has two human scFv binding arms, the HER3-binding scFv is shown to prevent HRG from attaching to HER3. The cell proliferation inhibition impact of MM-111 is amplified when cells are stimulated with HRG^[47]^. MM-111 can be utilized alone or combined with trastuzumab, lapatinib, and chemotherapeutic drugs in clinical studies of HER2+ cancers. Both MM-111 alone and in combination were shown to have anticancer effects (combination more effective than alone) in some HER2+ cancers^[3,99,100]^. Nevertheless, the phase II research with MM-111 in HER2+ gastroesophageal cancer (GOC) patients was ended prematurely because it was discovered that adding MM-111 to paclitaxel + trastuzumab resulted in significantly lower overall survival (OS) and progression-free survival (PFS)^[88]^.

#### Istiratumab (MM-141)

Tetravalent bsAb istiratumab has four high-affinity binding sites, two of which are dedicated to binding IGF-1R and the other two to binding HER3^[11,101]^. Istiratumab is a combination of a human IgG1 antibody against IGF-1R and two anti-HER3 scFvs attached to the carboxyl termini of the heavy chains^[101]^. The bsAb inhibits ligand binding and activation of both receptors, which in turn blocks the PI3K/AKT/mTOR pathway. Additionally, this antibody promotes the degradation of both receptors, including receptor dimers that consist of HER3 or IGF-1R^[89,101]^. This mechanism leads to a stronger inhibitory effect of MM-141 on cancer cell growth than the combination of HER3 and IGF-1R antibodies. In addition, MM-141 was demonstrated to increase the therapeutic efficacy of chemotherapeutics and a mTOR inhibitor, everolimus, via regulating HER3 and IGF-1R levels in models of pancreatic and ovarian cancer^[11,101,102]^. Nevertheless, the MM-141 adding istiratumab to standard chemotherapy showed no apparent clinical efficacy in patients with metastatic pancreatic cancer in a phase II study^[89]^.

#### Duligotuzumab (MEHD7945A, RG7597)

Duligotuzumab is a humanized bsAb IgG1 with two binding sites that can bind either the EGFR or HER3 subdomain III^[103]^. Duligotuzumab was shown to effectively block ligand-binding and downstream signaling of both EGFR and HER3 and mediate ADCC. When cancer development depends on EGFR and HER3, the antitumoral effect translates into enhanced growth suppression, especially in combination with chemotherapy in various xenograft models compared to monospecific therapies^[103]^. In a phase Ib study, duligotuzumab combined with cisplatin/5-fluorouracil or carboplatin/paclitaxel has shown encouraging results in patients with metastatic or recurrent HNSCC^[104]^. Duligotuzumab inhibited the proliferation of HNSCC and NSCLC cell lines that exhibited resistance to cetuximab and erlotinib in monotherapy or combination with cisplatin^[105,106]^. Duligotuzumab, in combination with trastuzumab, inhibited proliferation and migration and decreased apoptotic rate in HER2-overexpression gastric cancer cell lines^[107]^. Although duligotuzumab has exhibited cancer suppression efficacy in several cancer models, it is unsatisfactory in some phase I/II clinical trials^[90,91,108]^.

#### SI-B001

SI-B001, an IgG-(scFv)2 bsAb, targets EGFR and HER3. This bsAb comprises a full IgG with two heavy and two light chains, as well as two scFvs attached to the heavy or light chains’ C or N terminals^[3]^. SI-B001 has been shown to encourage antitumor efficacy along with acceptable tolerance in colon cancer, head and neck cancer and esophageal cancer models in preclinical studies. Now, it is studied in phase I and II clinical trials^[3,92]^.

### Antibody-drug conjugates

Antibody-drug conjugate (ADC) is composed of a monoclonal antibody that delivers and releases cytotoxic drug at the tumor site and should have strong target affinity, low cross-reactivity and low immunogenicity, a linker (including the cleavable linker and non-cleavable linker) which plays a role in binding the drug to the antibody and must be stable in circulation, and a cytotoxic drug (payload or warhead) which is the antitumor element of an ADC and should be non-immunogenic and non-toxic^[109]^. The mechanism of ADCs’ antitumor activity is generally thought to be: after ADC binds to the antigen, the ADC-antigen complex is internalized and translocated to the lysosome, where the ADC is destroyed to release the cytotoxic drug that kills cells^[110,111]^.

Antibody-drug conjugates use antibodies that are specific to antigens and have tumor selectivity and efficacy that standard medicines cannot match^[112]^. Recently, ADCs have been found to promote receptor endocytosis and destruction and cancer cell death^[113]^. According to the advantages of ADCs, HER3-targeting ADCs have emerged and shown potent antitumor efficacy in preclinical and clinical trials [Table 3].

**Table 3:** Summary of antibody-drug conjugates of HER3

**Compound**	**Components**	**Research status**	**Indication (cancer types)**	**Main side effects**	**Ref.**
U3-1402 (Patritumab deruxtecan, HER3-DXd)	Patritumab, cleavable linker and deruxtecan	Phase III trial	EGFR-mutated NSCLC, CRC, PRC and BC	Nausea, vomiting and alopecia	Hashimoto *et al*.^[110]^ Janne *et al*.^[114]^
EV20-Sap	EV20, cleavable linker and saporin	Preclinical	Melanoma	-	Capone *et al*.^[115]^
EV20/MMAF	EV20, non-cleavable linker and MMAF	Preclinical	Melanoma and BC	-	Capone *et al*.^[116]^
EV20‑sss‑vc/MMAF	EV20 variant, cleavable linker and MMAF	Preclinical	LC	-	D’Agostino *et al*.^[117]^
EV20/NMS-P945	EV20, cleavable linker and NMS-P945	Preclinical	HER3 + GC, OVC, HNSC, PAC, melanoma and PRC	-	Capone *et al*.^[118]^
MMAE-9F7-F11	MMAE, cleavable linker and 9F7-F11	Preclinical	PDAC	-	Bourillon *et al*.^[119]^
AMT-562	Ab562, T800 and exatecan	Preclinical	HER3 low PAC, ESC, COC and GC	-	Weng *et al*.^[120]^

HER: Human epidermal growth factor receptor; EGFR: epidermal growth factor receptor; NSCLC: non-small cell lung cancer; CRC: colorectal cancer; PRC: prostate cancer; BC: breast cancer; MMAF: monomethyl auristatin F; LC: liver cancer; GC: gastric cancer; OVC: ovarian cancer; HNSC: head and neck cancer; PAC: pancreatic cancer; PDAC: pancreatic ductal adenocarcinoma; ESC: esophagus cancer; COC: colon cancer.

#### U3-1402 (Patritumab deruxtecan, HER3-DXd)

U3-1402, also known as patritumab deruxtecan or HER3-DXd, is a HER3-directed ADC composed of patritumab, a cleavable maleimide-GGFG peptide linker, and a topoisomerase I inhibitor deruxtecan (DXd) payload^[110,121]^. U3-1402 exhibits HER3-specific affinity as well as efficient internalization into tumor cells and causes apoptosis in tumor cells through DNA damage via releasing DXd after linker cleavage^[110]^. U3-1402 downregulated HER3 and showed antigrowth activity in HER3-positive breast, prostate, and colorectal cancer^[16,110,122]^. In EGFR-TKI-resistant NSCLC models, U3-1402 was likewise efficacious both when used alone or in combination with an EGFR-TKI, and pretreatment with osimertinib improved the efficacy of U3-1402^[121,123,124]^. In addition, HER3-expressing cancers were sensitive to anti-PD-1 checkpoint blockage by U3-1402, indicating that U3-1402 and immunotherapy drugs can be combined for cancer treatment^[125]^. The efficacy and security of U3-1402 were tested in phase I/II clinical trials and showed that U3-1402 has an acceptable safety profile and a considerable objective response rate (ORR)^[126-128]^. Therefore, U3-1402 is an excellent treatment option for patients with HER3+ cancers and is undergoing a phase III trial in EGFR-mutated NSCLC^[127]^.

#### EV20-derived HER3-specific ADCs

EV20 is an IgG1 humanized monoclonal antibody targeting HER3, and it is quickly and effectively internalized by HER3-positive cancer cells. The latter characteristic makes EV20 a strong contender for ADC synthesis^[58]^. EV20-Sap is a new ADC created by chemically crosslinking EV20 to the ribosome-inactivating protein saporin. EV20-Sap was demonstrated to display a robust, selective, and target-dependent lethal function in HER3-expressing cancer cell lines, and elevated expression levels of HER3 in cancer cells were associated with efficient internalization, effectiveness, and cytotoxic effects. In melanoma cells, the activity of EV20-Sap was matched to HER3 expression and unaffected by NRG-1β ligand *in vitro*; moreover, it was also influential in minimizing the size and the number of lung metastases *in vivo*^[115]^.

EV20/monomethyl auristatin F (MMAF) consists of the humanized anti-HER3 antibody EV20 coupled with a non-cleavable linker to MMAF^[129]^. EV20/MMAF had the ability to target melanoma cells and breast cancer cells specifically and efficiently, and the cell killing activity of ADCs was unaffected by the cells’ BRAF status^[129]^. EV20/MMAF also exhibited a robust and effective antitumoral effect in HER2+ melanoma and breast cancer models, including cells resistant to anti-HER2 therapeutics^[129,130]^. Additionally, EV20/MMAF has highly stable, well-tolerated, and low toxicity profiles^[129]^.

EV20-sss-vc/MMAF was generated by MMAF site-specifically connected to an engineered variant of EV20 through a vc cleavable linker. EV20-sss-vc/MMAF showed higher cell killing activity and tumor growth-inhibiting activity than clinically approved T-DM1 and EV20/MMAF in liver cancer cell lines^[117]^.

EV20/NMS-P945 was generated by EV20 connected to a duocarmycin-like thienoindole derivative NMS-P528, applying a peptide cleavable linker and a self-immolating spacer. EV20/NMS-P945 showed robust and target-dependent anticancer activity in HER3-positive gastric, pancreatic, ovarian, melanoma, and prostatic cancer. The ADC was well tolerated, had high stability, and held an excellent pharmacokinetic profile *in vivo* in monkey plasma^[118]^.

#### MMAE-9F7-F11

MMAE-9F7-F11, a HER3-targeting ADC, was generated by antibody 9F7-F11 coupled with a cleavable linker maleimidocaproyl-valine-citrulline-p- aminobenzyloxycarbonyl (MC-vc-PAB) to monomethyl auristatin E (MMAE) which is a radiosensitizer. MMAE-9F7-F11 triggered cell cycle arrest in G2/M and enhanced radiosensitivity in pancreatic ductal adenocarcinoma (PDAC). Additionally, MMAE-9F7-F11 restricted the compensatory activation of AKT signaling after irradiation. In a pancreatic cancer mouse model, MMAE-9F7-F11 with radiation therapy improved OS *in vivo*^[119]^.

#### AMT-562

AMT-562, a new ADC, was synthesized by an anti-HER3 antibody Ab562 and a PABC spacer (T800) to link to exatecan. AMT-562 has more profound and long-lasting anticancer responses in low HER3 expression cell lines and pancreatic, esophagus, colon, and gastric cancer models. AMT-562 has great pharmacokinetic and safety profiles, and its wider therapeutic window allows it to conquer resistance to elicit higher percentages and longer-lasting responses in malignancies resistant to U3-1402^[120]^. Based on the superiority of AMT-562 in cancer treatment, it is starting to be studied in clinical malignancies.

### Pan-HER strategies

Owing to many cancers expressing more than one HER family receptor, inhibitors targeting multiple HER receptors at different points are more effective than those targeting only one receptor. Pan-HER inhibitors are either TKIs or a combination of antibodies that target non-overlapping epitopes on EGFR, HER2, and HER3^[131]^. Due to the variety and flexibility of tumors, pan-HER are shown to be superior to single mAbs in postponing both inherent and acquired resistance^[132,133]^. Next, we mainly focused on pan-HER strategies targeting HER3.

#### AZD8931 (sapitinib)

AZD8931 is a powerful and reversible tyrosine kinase inhibitor of EGFR, HER2, and HER3 signaling. *In vitro*, in comparison to alternative EGFR inhibitors like lapatinib or gefitinib, AZD8931 was found to be substantially more effective against EGFR, HER2, and HER3 signaling. *In vivo*, AZD8931 was demonstrated to hold stronger anticancer capacity in a variety of xenografted models compared to lapatinib or gefitinib^[134]^. AZD8931 combined with chemotherapy was studied in some cancer models and exhibited encouraging outcomes. AZD8931 markedly reduced cell growth and triggered human inflammatory breast cancer (IBC) cell apoptosis. In orthotopic IBC models, the combination of paclitaxel and AZD8931 proved to be more successful in slowing tumor growth than either drug alone^[135]^. In phase I/II research, high-dose pulsed AZD8931 combined with irinotecan/5-FU chemotherapy had acceptable toxicity in metastatic colorectal cancer (CRC)^[136]^. Oxaliplatin + capecitabine plus AZD8931 had a tolerable safety profile in oesophagogastric cancer^[137]^. So AZD8931 is a promising therapeutic agent that provides a potential option for cancer treatment.

#### Sym013

Sym013 is a blend of six humanized IgG1 mAbs that target non-overlapping epitopes on domain III of EGFR, domain III and IV of HER2, and domain I of HER3 in a balanced ratio. Binding to non-overlapping epitopes induces receptor internalization and degradation, which prevents downstream signaling^[132,138]^. *In vitro* studies demonstrated that different cancer cell lines, including those with inherent and acquired resistance to HER-targeted treatments, successfully slowed down their ability to proliferate by Sym013. Sym013 also significantly inhibited tumor growth in various xenograft models, including pancreatic, squamous lung, ovarian, breast, and colorectal cancer models^[132,139-141]^. Despite starting phase I/II research with Sym013, clinical development was abandoned due to an unidentified toxicity profile^[142]^.

### Vaccinations of HER3

As an alternative to mAbs, vaccinations can induce polyclonal antibodies that can recognize the receptor, prevent its phosphorylation, facilitate the internalization and destruction of the receptor, and inhibit cancer proliferation. Additionally, vaccines trigger enduring antitumor immune reactions that can be periodically strengthened^[143]^. As a result, vaccinations could be an effective way for cancer treatment. Until now, several vaccinations targeting HER3 have been created, and two of them are tested in clinical trials.

#### HER3-targeted vaccinations under clinical development

pING-hHER3FL is a circular fragment of DNA that encodes the entire HER3 protein. At present, pING-hHER3FL is used in a phase I study as immunotherapeutics to target HER3-positive malignancies^[3]^.

NCT04348747 uses dendritic cell vaccines targeting HER2/HER3, and the combination with pembrolizumab can strengthen the immune system and enhance the tumor immune responses, which is favorable to shrinking cancer. This vaccination was effective in treating TNBC or HER2+ brain metastasis BC in a phase II trial study^[3]^.

#### HER3-targeted vaccinations in preclinical studies

Ad-HER3-FL (Ad-HER3) was created with an adenoviral vector encoding full-length human HER3 receptor. Ad-HER3 produced potent antitumor T-cell responses as well as anti-HER3 antibodies, both of which effectively combated BC^[143,144]^. Moreover, Ad-HER3-FL combined with dual PD-1/PD-L1 and CTLA4 showed higher efficacy than the vaccination alone^[144]^.

A study evaluated novel HER3 peptide epitopes that may be B cell epitopes, including residues 99-122, 140-162, 237-269, and 461-479 of the HER3-ECD. It has the ability to initiate immunotherapy for HER3-positive tumors^[145]^. Among the four HER3 epitopes, 237-269 and 461-479 markedly prevented the growth of xenografts deriving from both pancreatic and breast cancers. In addition, combining HER3 (461-471) epitope vaccination antibodies and peptide mimics with HER2 (266-296), HER2 (597-626), HER1 (418-435), and IGF-1R (56-81) vaccine antibodies and peptide mimics improved anticancer effectiveness^[145,146]^.

## POTENTIAL APPROACHES TARGETING HER3 FOR CANCER THERAPY

**Figure 3:**
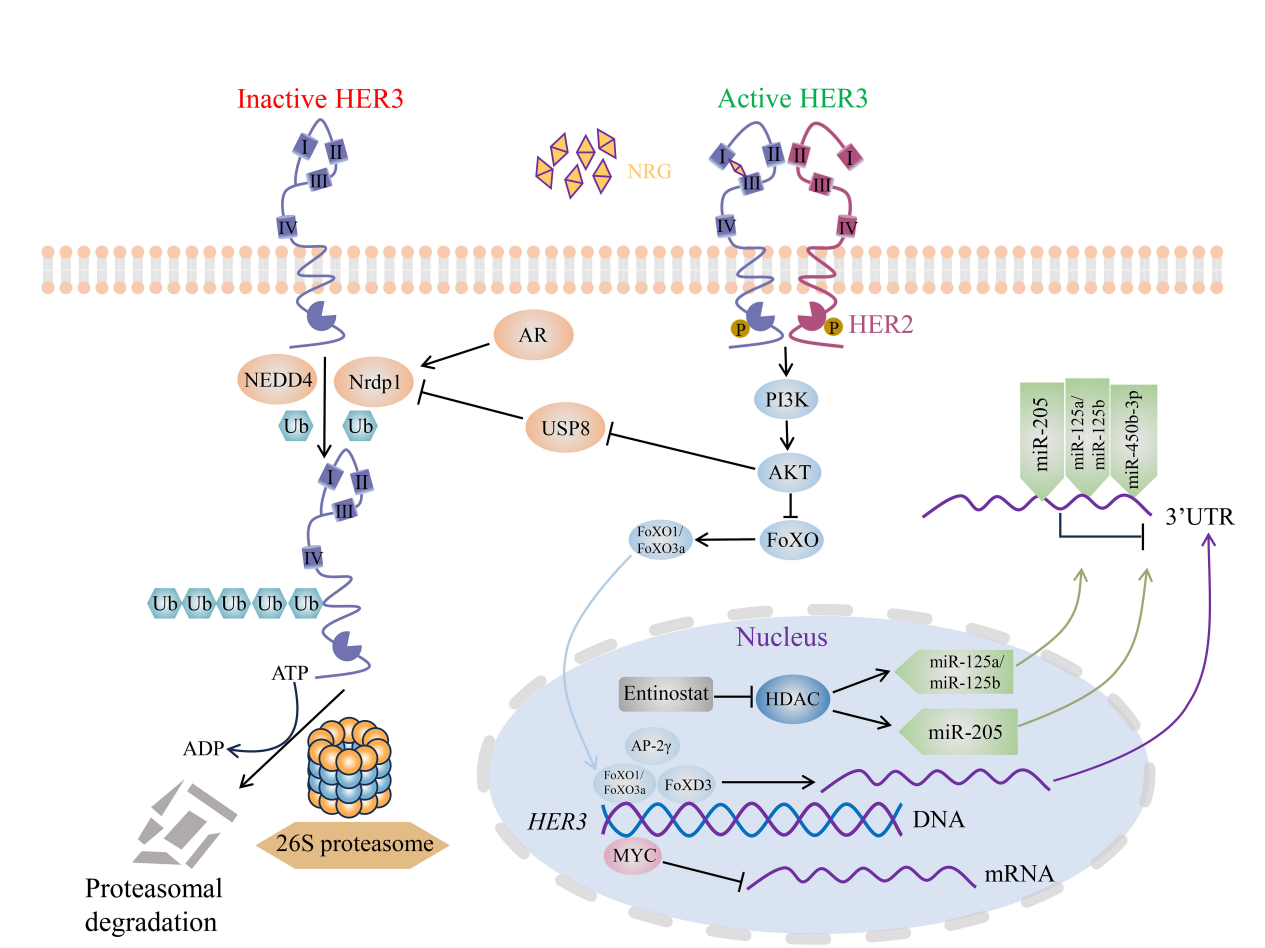
Potential approaches for HER3-targeted cancer therapy. Nrdp1 and NEDD4 are E3 ubiquitin ligases of HER3, which promote the ubiquitination of HER3 and 26S proteasome degradation, and subsequently downregulate the protein level of HER3. The activated AR acts as a transcription factor facilitating Nrdp1 transcription. Moreover, AKT inhibits the deubiquitinating enzyme USP8, while USP8 downregulates Nrdp1 levels. In addition, AKT inhibits the expression of FoXO transcription factor, and FoXO family transcription factors FoXO1 and FoXO3a can target the HER3 promoter to promote HER3 transcription. FoXD3 and AP-2γ are also transcriptional activators of HER3, while MYC is a transcriptional suppressor of HER3. Epigenetic inhibitors entinostat inhibits HDAC, HDAC can activate miR-125a, miR-125b, and miR-205; these three miRNAs can complement with 3’UTR of HER3 mRNA, subsequently downregulate HER3 levels. Furthermore, miR-450b-3p can also control HER3 levels. HER: Human epidermal growth factor receptor; NEDD4: neural precursor cell expressed developmentally down-regulated 4; Nrdp1: neuregulin receptor degradation protein 1; Ub: ubiquitin; ATP: adenosine triphosphate; ADP: adenosine diphosphate; NRG: neuregulin; AR: androgen receptor; USP8: ubiquitin-specific protease 8; PI3K: phosphatidylinositide 3-kinases; AKT: also named PKB (protein kinase B); FoXO: forkhead box O; HDAC: histone deacetylase; AP-2γ: activator protein-2γ; MYC: myelocytomatosis; 3’UTR: 3’-untranslated region.

Because of the significance of HER3 in the progression of multiple malignancies and the occurrence of drug resistance and toxicity, identifying novel approaches provides more options for HER3+ cancer therapy. In addition to the therapies mentioned above that effectively target HER3 for cancer treatment, some molecules have emerged in recent years and can become future drug design targets for HER3, including E3 ubiquitin ligases, microRNAs, and transcription factors [Figure 3].

### E3 ubiquitin ligases of HER3

It has been demonstrated that ubiquitination controls the levels of EGFR family receptors by sending them to be degraded by proteasomes or lysosomes^[147]^. The mechanism of ubiquitination is that ubiquitin (Ub) is activated by ubiquitin-activating enzyme (E1) and is then transported to ubiquitin-conjugating enzyme (E2), and ubiquitin ligase (E3) binds the substrate and catalyzes the Ub transporting to the substrate. Finally, the substrate with Ub is detected and destroyed by the 26S proteasome^[148]^. Previous studies showed that E3 ubiquitin ligase plays a prominent role in regulating EGFR family receptors’ levels. For example, E3 ubiquitin ligase Cbl interacts with EGFR to induce its lysosomal degradation, and CHIP interacts with HER2 to facilitate its degradation^[149,150]^. WWP1 ubiquitinates HER4 to mediate both proteasomal and lysosomal degradation^[151]^. There are also some E3 ubiquitin ligases targeting HER3 to control its levels, which are essential for maintaining homeostasis and can be the targets for drug design or prognostic markers for HER3+ cancers.

#### Nrdp1

Nrdp1 (neuregulin receptor degradation protein 1), a RING E3 ubiquitin ligase, can ubiquitinate and lead to proteasomal degradation of HER3 independent of NRG1 (neuregulin-1) activation^[152]^. A growing body of studies suggested that Nrdp1 ubiquitinates and degrades HER3, influencing cancer cell growth and progression. For instance, Nrdp1 ubiquitinated and decreased the level of ErbB3, subsequently suppressing glioma cell migration and invasion^[153]^. Nrdp1-mediated ErbB3 degradation inhibited cellular proliferation and motility, while Nrdp1 depletion in BC could accelerate cancer growth by enhancing ErbB2/ErbB3 signaling^[154]^. Further studies need to investigate whether the level of the Nrdp1 can serve as a biomarker of HER3-targeted therapy.

#### NEDD4

NEDD4 (neural precursor cell expressed developmentally down-regulated 4), a HECT E3 ubiquitin ligase, was identified as an E3 ubiquitin ligase of HER3. The C-terminal tail of HER3 interacts with the WW domains of NEDD4, and the interaction was independent of NRG-1. In breast and prostate cancer models, the knockdown of NEDD4 boosted HER3 expression, HER3 signaling, cell proliferation, and cancer progression. Moreover, upregulating HER3 expression sensitized cancer cells for growth suppression by an anti-HER3 mAb^[155]^. One study discovered that the nonreceptor tyrosine kinases PYK2/FAK interacted with NEDD4 and HER3, interfering with NEDD4-HER3 interaction, while NDRG1 (N-myc downstream regulated 1 gene) facilitated HER3 and NEDD4 binding. This finding suggests that the PYK2-NDRG1-NEDD4 loop is crucial for HER3 receptor degradation, HER3 downstream signaling activation, and TNBC therapy^[156]^. According to the negative correlation between NEDD4 and HER3, NEDD4 can be a potential target for HER3-positive cancer treatment.

### microRNAs of HER3

microRNA (miRNA), a category of highly conserved small noncoding RNA, binds to the 3’UTR of mRNA and regulates gene expression at the posttranscriptional level^[157]^. Undoubtedly, miRNA can be involved in the development of multiple diseases. An expanding body of studies suggests that miRNA expression is significantly correlated with tumorigenesis, and it can act as both the cancer promoter and cancer suppressor^[158]^. As the pivotal role of HER3 in cancer development, we mainly focused on miRNAs of HER3, which can be a novel strategy for HER3+ cancer treatment.

#### miR-205

miR-205 was initially found to be connected to the deficiency of vascular invasion in BC. The expression of miR-205 is limited in normal myoepithelial cells, while it is decreased or absent in cancer cells^[159]^. It was discovered that HER3 was one of the presumptive targets of miR-205; miR-205 bound to the 3’UTR of HER3 and VEGF-A mRNA and then downregulated their levels, which resulted in the inhibition of breast cancer cell growth^[160]^. Further, miR-205 targeted HER3 and suppressed the growth, metastasis, and invasion, and enhanced the chemosensitivity of human nasopharyngeal carcinoma cells^[161]^.

#### miR-125a and miR-125b

miR-125a and miR-125b can target both HER2 and HER3, decreasing their transcript and protein levels in breast cancer cells. Functionally, miR-125a- or miR-125b-overexpressing cancer cells markedly suppressed breast cancer cell migration and invasion, as well as enhancing trastuzumab and paclitaxel efficacy, but only had minimal effects in mammary epithelial cells^[162,163]^. Moreover, miR-125a and miR-205 co-expression had stronger effects in inhibiting HER3 than either miRNA alone in HER2+ BC cell lines^[163]^.

#### miR-450b-3p

miR-450b-3p binds to the HER3 mRNA to suppress HER3 expression and downstream signaling, subsequently inhibiting breast cancer cell growth and enhancing cancer cell sensitivity to trastuzumab and doxorubicin^[164]^. Therefore, the miR-450b-3p level may be regarded as a prognostic biomarker of HER3+ BC patients.

### Transcription factors of HER3

Transcription factors are essential for controlling gene expression and multiple biological processes, as well as maintaining cellular homeostasis. Once they are misregulated, they can lead to a variety of diseases, including cancer^[165]^. Based on the role of transcription factors in cancers, transcription factors are classified into two groups: pro-cancer transcription factors and anticancer transcription factors^[166]^. Given the carcinogenic role of HER3 in a variety of cancers, finding transcription factors of HER3 is significant for creating new therapeutic methods.

#### MYC

MYC is an oncogenic transcription factor and a potent driver of various human cancers^[167]^. MYC was found to be a negative transcription factor of ERBB2^[168]^. In addition, the drop in MYC levels caused by MEK inhibitor was followed by an increase in HER2 and HER3 mRNA and protein levels in KRAS mutant lung and colon cancer cell lines. This indicates that MYC is not only a transcriptional repressor of HER2 but also of HER3^[169]^.

#### FoXO1 and FoXO3a

Forkhead box O (FoXO) proteins are transcription factors with four members, including FoXO1, FoXO3a, FoXO4, and FoXO6 in mammals. Functionally, they are involved in regulating cell growth, apoptosis, DNA repair, tumor suppression, and metabolism^[170]^. FoXOs generally display antitumor effects and are inactivated in a variety of human malignancies^[170]^. Thus, FoXOs have functions that regulate HER3, which is not surprising. HER2 inhibited by lapatinib resulted in FoXO3a-dependent upregulation of HER3 mRNA and protein, which partially maintained phosphorylated AKT (p-AKT) and limited the antitumor action of lapatinib^[171]^. In addition, the knockdown of FoXO1 and FoXO3a transcription factors led to HER3 mRNA induction failure upon inhibition of PI3K in HER2+ breast cancer cell lines^[172]^. As a result, FoXO1 and FoXO3a are both transcription factors of HER3 and are involved in the PI3K/AKT signaling pathway.

#### FoXD3

Forkhead box D3 (FoXD3) is a stem cell/pluripotency transcription factor that can be triggered upon BRAF/MEK pathway inhibition in mutant BRAF melanomas^[173]^. SOX10 binds to the promoter of FoXD3, activates the transcription of FoXD3, and upregulates FoXD3 levels^[174]^. FoXD3, a transcription factor of HER3, can upregulate HER3 and increase the resistance to rapidly accelerated fibrosarcoma (RAF) inhibitors by activating PI3K/AKT signaling in melanoma cell lines and mouse xenograft models^[175]^. Except for FoXO1 and FoXO3a, FoXD3 is also a transcription factor of HER3 and has a vital role in anticancer therapy.

#### AP-2γ

Activator protein-2 (AP-2), a transcriptional regulating factor, is composed of five members: AP-2α, AP-2β, AP-2γ, AP-2δ, and AP-2ε. AP-2 is not only required for normal development but has also been linked to tumorigenesis^[176]^. In human mammary epithelial and fibroblast cell lines, AP-2γ was positively linked to endogenous ErbB3 mRNA levels instead of AP-2α and AP-2β. In addition, the AP-2α deletion mutant, which can saliently interfere with the transcriptional activation function of each AP-2 member, not only inhibited the ErbB3 promoter activity but also suppressed ErbB3 transcription in the ErbB3-overexpression breast cancer cell lines^[177]^.

## CONCLUSION

HER3 is upregulated in numerous cancers, and many studies have shown that HER3 promotes cancer progression and drug resistance. Due to its interaction with PI3K, HER3 is crucial in developing drug resistance via the PI3K/AKT signaling pathway. HER3 antibodies can be involved in cancer treatment in combination with compounds targeting EGFR and HER2, endocrine therapeutic agents, and chemotherapeutic agents, but led to resistance to some therapies, including targeted therapy, chemotherapy, radiotherapy, and hormone therapy^[8,178]^. The primary mechanism of drug resistance is that different agents can activate EGFR, HER2, and HER3. The activated HER3 can form heterodimers with EGFR and HER2, allowing HER3 to activate PI3K/AKT and MEK/MAPK pathways. As a result, finding the mechanism of drug resistance and developing new drugs are crucial for conquering resistance and improving treatment efficacy.

Over the years, much of the research on HER3-targeted therapies mainly focuses on the development of antibodies. Monoclonal antibodies are the most abundant agents binding to HER3-ECD, and some have undergone clinical phase I/II/III evaluation, such as patritumab, seribantumab, and lumretuzumab. Although the safety profile of these antibodies in clinical trials is acceptable, their effectiveness is unsatisfactory, and the development of most of them is halted in clinical trials. In order to increase the effectiveness of treatment, bispecific antibodies, antibody-drug conjugates, pan-HER strategies, and HER3 vaccinations have been created and measured in phase I/II trials in a variety of cancers. In addition, other novel approaches, including siRNA, antisense oligonucleotide EZN-3920^[179]^, and bispecific ligand trap RB200^[180]^, exerted effective anticancer efficacy in human cancers. Some molecules, including E3 ubiquitin ligases, miRNAs, and transcription factors, can serve as potential targets for drug design of HER3. According to a number of studies, cancers with high levels of NRG1 expression before antibody treatment may respond better to HER3 antibodies that do not compete with the NRG binding site^[181,182]^. Therefore, NRG, as a biomarker, could be utilized to predict HER3 mAbs response in the future.

## DECLARATIONS

### Copyright

© The Author(s) 2024.

## References

[1] Sung H, Ferlay J, Siegel RL (2021). Global cancer statistics 2020: GLOBOCAN estimates of incidence and mortality worldwide for 36 cancers in 185 countries. CA Cancer J Clin.

[2] Lepeltier E, Rijo P, Rizzolio F (2020). Nanomedicine to target multidrug resistant tumors. Drug Resist Updat.

[3] Gandullo-Sánchez L, Ocaña A, Pandiella A (2022). HER3 in cancer: from the bench to the bedside. J Exp Clin Cancer Res.

[4] Uliano J, Corvaja C, Curigliano G, Tarantino P (2023). Targeting HER3 for cancer treatment: a new horizon for an old target. ESMO Open.

[5] Appert-Collin A, Hubert P, Crémel G, Bennasroune A (2015). Role of ErbB receptors in cancer cell migration and invasion. Front Pharmacol.

[6] Diwanji D, Trenker R, Thaker TM (2021). Structures of the HER2-HER3-NRG1β complex reveal a dynamic dimer interface. Nature.

[7] Montero JC, Rodríguez-Barrueco R, Ocaña A, Díaz-Rodríguez E, Esparís-Ogando A, Pandiella A (2008). Neuregulins and cancer. Clin Cancer Res.

[8] Haikala HM, Jänne PA (2021). Thirty years of HER3: from basic biology to therapeutic interventions. Clin Cancer Res.

[9] Frazier NM, Brand T, Gordan JD, Grandis J, Jura N (2019). Overexpression-mediated activation of MET in the Golgi promotes HER3/ERBB3 phosphorylation. Oncogene.

[10] Kunii K, Davis L, Gorenstein J (2008). FGFR2-amplified gastric cancer cell lines require FGFR2 and Erbb3 signaling for growth and survival. Cancer Res.

[11] Camblin AJ, Tan G, Curley MD (2019). Dual targeting of IGF-1R and ErbB3 as a potential therapeutic regimen for ovarian cancer. Sci Rep.

[12] Shi F, Telesco SE, Liu Y, Radhakrishnan R, Lemmon MA (2010). ErbB3/HER3 intracellular domain is competent to bind ATP and catalyze autophosphorylation. Proc Natl Acad Sci U S A.

[13] Steinkamp MP, Low-Nam ST, Yang S, Lidke KA, Lidke DS, Wilson BS (2014). erbB3 is an active tyrosine kinase capable of homo- and heterointeractions. Mol Cell Biol.

[14] Majumder A (2023). HER3: toward the prognostic significance, therapeutic potential, current challenges, and future therapeutics in different types of cancer. Cells.

[15] Xue C, Liang F, Mahmood R (2006). ErbB3-dependent motility and intravasation in breast cancer metastasis. Cancer Res.

[17] Scharpenseel H, Hanssen A, Loges S (2019). EGFR and HER3 expression in circulating tumor cells and tumor tissue from non-small cell lung cancer patients. Sci Rep.

[18] Lédel F, Hallström M, Ragnhammar P, Öhrling K, Edler D (2014). HER3 expression in patients with primary colorectal cancer and corresponding lymph node metastases related to clinical outcome. Eur J Cancer.

[19] Ueno Y, Sakurai H, Tsunoda S (2008). Heregulin-induced activation of ErbB3 by EGFR tyrosine kinase activity promotes tumor growth and metastasis in melanoma cells. Int J Cancer.

[20] Sheng Q, Liu X, Fleming E (2010). An activated ErbB3/NRG1 autocrine loop supports in vivo proliferation in ovarian cancer cells. Cancer Cell.

[21] Wang L, Yuan H, Li Y, Han Y (2014). The role of HER3 in gastric cancer. Biomed Pharmacother.

[22] Liles JS, Arnoletti JP, Tzeng CW (2010). ErbB3 expression promotes tumorigenesis in pancreatic adenocarcinoma. Cancer Biol Ther.

[23] Wang Z, Goto Y, Allevato MM (2021). Disruption of the HER3-PI3K-mTOR oncogenic signaling axis and PD-1 blockade as a multimodal precision immunotherapy in head and neck cancer. Nat Commun.

[24] Liu X, Liu S, Lyu H, Riker AI, Zhang Y, Liu B (2019). Development of effective therapeutics targeting HER3 for cancer treatment. Biol Proced Online.

[25] Liu R, Chen Y, Liu G (2020). PI3K/AKT pathway as a key link modulates the multidrug resistance of cancers. Cell Death Dis.

[26] Lee L, Ramos-Alvarez I, Moody TW, Mantey SA, Jensen RT (2020). Neuropeptide bombesin receptor activation stimulates growth of lung cancer cells through HER3 with a MAPK-dependent mechanism. Biochim Biophys Acta Mol Cell Res.

[27] Lee S, Rauch J, Kolch W (2020). Targeting MAPK signaling in cancer: mechanisms of drug resistance and sensitivity. Int J Mol Sci.

[28] Huang XL, Khan MI, Wang J (2021). Role of receptor tyrosine kinases mediated signal transduction pathways in tumor growth and angiogenesis - New insight and futuristic vision. Int J Biol Macromol.

[29] Erjala K, Sundvall M, Junttila TT (2006). Signaling via ErbB2 and ErbB3 associates with resistance and epidermal growth factor receptor (EGFR) amplification with sensitivity to EGFR inhibitor gefitinib in head and neck squamous cell carcinoma cells. Clin Cancer Res.

[30] Huang X, Gao L, Wang S (2010). Heterotrimerization of the growth factor receptors erbB2, erbB3, and insulin-like growth factor-i receptor in breast cancer cells resistant to herceptin. Cancer Res.

[31] Yang L, Li Y, Shen E (2017). NRG1-dependent activation of HER3 induces primary resistance to trastuzumab in HER2-overexpressing breast cancer cells. Int J Oncol.

[32] Ye P, Wang Y, Li R, Chen W, Wan L, Cai P (2022). The HER family as therapeutic targets in colorectal cancer. Crit Rev Oncol Hematol.

[33] Tao JJ, Castel P, Radosevic-Robin N (2014). Antagonism of EGFR and HER3 enhances the response to inhibitors of the PI3K-Akt pathway in triple-negative breast cancer. Sci Signal.

[34] Zhang Y, Liang S, Xiao B (2022). MiR-323a regulates ErbB3/EGFR and blocks gefitinib resistance acquisition in colorectal cancer. Cell Death Dis.

[35] Engelman JA, Zejnullahu K, Mitsudomi T (2007). MET amplification leads to gefitinib resistance in lung cancer by activating ERBB3 signaling. Science.

[36] Wang S, Huang X, Lee CK, Liu B (2010). Elevated expression of erbB3 confers paclitaxel resistance in erbB2-overexpressing breast cancer cells via upregulation of Survivin. Oncogene.

[37] Knuefermann C, Lu Y, Liu B (2003). HER2/PI-3K/Akt activation leads to a multidrug resistance in human breast adenocarcinoma cells. Oncogene.

[38] Bezler M, Hengstler JG, Ullrich A (2012). Inhibition of doxorubicin-induced HER3-PI3K-AKT signalling enhances apoptosis of ovarian cancer cells. Mol Oncol.

[39] He G, Di X, Yan J, Zhu C, Sun X, Zhang S (2018). Silencing human epidermal growth factor receptor-3 radiosensitizes human luminal A breast cancer cells. Cancer Sci.

[40] Shou J, Massarweh S, Osborne CK (2004). Mechanisms of tamoxifen resistance: increased estrogen receptor-HER2/neu cross-talk in ER/HER2-positive breast cancer. J Natl Cancer Inst.

[41] Liu B, Ordonez-Ercan D, Fan Z, Edgerton SM, Yang X, Thor AD (2007). Downregulation of erbB3 abrogates erbB2-mediated tamoxifen resistance in breast cancer cells. Int J Cancer.

[42] Papadimitropoulou A, Vellon L, Atlas E (2020). Heregulin drives endocrine resistance by altering IL-8 expression in ER-positive breast cancer. Int J Mol Sci.

[43] Frogne T, Benjaminsen RV, Sonne-Hansen K (2009). Activation of ErbB3, EGFR and Erk is essential for growth of human breast cancer cell lines with acquired resistance to fulvestrant. Breast Cancer Res Treat.

[44] Jathal MK, Chen L, Mudryj M, Ghosh PM (2011). Targeting ErbB3: the new RTK(id) on the prostate cancer block. Immunol Endocr Metab Agents Med Chem.

[45] Zhang Z, Karthaus WR, Lee YS (2020). Tumor microenvironment-derived NRG1 promotes antiandrogen resistance in prostate cancer. Cancer Cell.

[46] Drago JZ, Ferraro E, Abuhadra N, Modi S (2022). Beyond HER2: targeting the ErbB receptor family in breast cancer. Cancer Treat Rev.

[47] Malm M, Frejd FY, Ståhl S, Löfblom J (2016). Targeting HER3 using mono- and bispecific antibodies or alternative scaffolds. MAbs.

[49] Sequist LV, Gray JE, Harb WA (2019). Randomized phase II trial of seribantumab in combination with erlotinib in patients with EGFR wild-type non-small cell lung cancer. Oncologist.

[50] Cleary JM, Mcree AJ, O’neil BH (2014). A phase 1 study of MM-121 (a fully human monoclonal antibody targeting the epidermal growth factor receptor family member ErbB3) in combination with cetuximab and irinotecan in patients with advanced cancers. J Clin Oncol.

[51] Cejalvo JM, Jacob W, Fleitas Kanonnikoff T (2019). A phase Ib/II study of HER3-targeting lumretuzumab in combination with carboplatin and paclitaxel as first-line treatment in patients with advanced or metastatic squamous non-small cell lung cancer. ESMO Open.

[52] Mirschberger C, Schiller CB, Schräml M (2013). RG7116, a therapeutic antibody that binds the inactive HER3 receptor and is optimized for immune effector activation. Cancer Res.

[53] Jhaveri K, Drago JZ, Shah PD (2021). A phase I study of alpelisib in combination with trastuzumab and LJM716 in patients with PIK3CA-mutated HER2-positive metastatic breast cancer. Clin Cancer Res.

[54] Hong M, Yoo Y, Kim M (2021). A novel therapeutic anti-ErbB3, ISU104 Exhibits potent antitumorigenic activity by inhibiting ligand binding and ErbB3 heterodimerization. Mol Cancer Ther.

[55] Duvvuri U, George J, Kim S (2019). Molecular and clinical activity of CDX-3379, an anti-ErbB3 Monoclonal antibody, in head and neck squamous cell carcinoma patients. Clin Cancer Res.

[56] Meetze K, Vincent S, Tyler S (2015). Neuregulin 1 expression is a predictive biomarker for response to AV-203, an ERBB3 inhibitory antibody, in human tumor models. Clin Cancer Res.

[57] Menke-van der Houven van Oordt CW, McGeoch A, Bergstrom M (2019). Immuno-PET imaging to assess target engagement: experience from ^89^Zr-Anti-HER3 mAb (GSK2849330) in patients with solid tumors. J Nucl Med.

[58] Sala G, Rapposelli IG, Ghasemi R, Consorzio Interuniversitario Nazionale per la Bio-Oncologia (CINBO) (2013). EV20, a novel anti-ErbB-3 humanized antibody, promotes ErbB-3 down-regulation and inhibits tumor growth in vivo. Transl Oncol.

[59] Wang Q, Zhang X, Shen E (2016). The anti-HER3 antibody in combination with trastuzumab exerts synergistic antitumor activity in HER2-positive gastric cancer. Cancer Lett.

[60] Lazrek Y, Dubreuil O, Garambois V (2013). Anti-HER3 domain 1 and 3 antibodies reduce tumor growth by hindering HER2/HER3 dimerization and AKT-induced MDM2, XIAP, and FoxO1 phosphorylation. Neoplasia.

[61] Turowec JP, Lau EWT, Wang X (2019). Functional genomic characterization of a synthetic anti-HER3 antibody reveals a role for ubiquitination by RNF41 in the anti-proliferative response. J Biol Chem.

[62] Schmitt LC, Rau A, Seifert O (2017). Inhibition of HER3 activation and tumor growth with a human antibody binding to a conserved epitope formed by domain III and IV. MAbs.

[63] Thakkar D, Sancenon V, Taguiam MM (2020). 10D1F, an anti-HER3 antibody that uniquely blocks the receptor heterodimerization interface, potently inhibits tumor growth across a broad panel of tumor models. Mol Cancer Ther.

[64] LoRusso P, Jänne PA, Oliveira M (2013). Phase I study of U3-1287, a fully human anti-HER3 monoclonal antibody, in patients with advanced solid tumors. Clin Cancer Res.

[65] Shimizu T, Yonesaka K, Hayashi H (2017). Phase 1 study of new formulation of patritumab (U3-1287) Process 2, a fully human anti-HER3 monoclonal antibody in combination with erlotinib in Japanese patients with advanced non-small cell lung cancer. Cancer Chemother Pharmacol.

[66] Yonesaka K, Hirotani K, Kawakami H (2016). Anti-HER3 monoclonal antibody patritumab sensitizes refractory non-small cell lung cancer to the epidermal growth factor receptor inhibitor erlotinib. Oncogene.

[67] Kawakami H, Okamoto I, Yonesaka K (2014). The anti-HER3 antibody patritumab abrogates cetuximab resistance mediated by heregulin in colorectal cancer cells. Oncotarget.

[68] Li C, Brand TM, Iida M (2013). Human epidermal growth factor receptor 3 (HER3) blockade with U3-1287/AMG888 enhances the efficacy of radiation therapy in lung and head and neck carcinoma. Discov Med.

[69] Denlinger CS, Keedy VL, Moyo V, MacBeath G, Shapiro GI (2021). Phase 1 dose escalation study of seribantumab (MM-121), an anti-HER3 monoclonal antibody, in patients with advanced solid tumors. Invest New Drugs.

[70] Rathore M, Zhang W, Wright M (2022). Liver endothelium promotes HER3-mediated cell survival in colorectal cancer with wild-type and mutant KRAS. Mol Cancer Res.

[71] Thavaneswaran S, Chan WY, Asghari R (2022). Clinical response to seribantumab, an anti-human epidermal growth factor receptor-3 immunoglobulin 2 monoclonal antibody, in a patient with metastatic pancreatic ductal adenocarcinoma harboring an NRG1 fusion. JCO Precis Oncol.

[72] Odintsov I, Lui AJW, Sisso WJ (2021). The anti-HER3 mAb seribantumab effectively inhibits growth of patient-derived and isogenic cell line and xenograft models with oncogenic *NRG1* fusions. Clin Cancer Res.

[73] Rendell A, Thomas-Bland I, McCuish L, Taylor C, Binju M, Yu Y (2022). Targeting tyrosine kinases in ovarian cancer: small molecule inhibitor and monoclonal antibody, where are we now?. Biomedicines.

[74] Steele TM, Tsamouri MM, Siddiqui S (2023). Cisplatin-induced increase in heregulin 1 and its attenuation by the monoclonal ErbB3 antibody seribantumab in bladder cancer. Sci Rep.

[75] Abramson VG, Supko JG, Ballinger T (2017). Phase Ib study of safety and pharmacokinetics of the PI3K inhibitor SAR245408 with the HER3-neutralizing human antibody SAR256212 in patients with solid tumors. Clin Cancer Res.

[76] Huang J, Wang S, Lyu H (2013). The anti-erbB3 antibody MM-121/SAR256212 in combination with trastuzumab exerts potent antitumor activity against trastuzumab-resistant breast cancer cells. Mol Cancer.

[77] Liles JS, Arnoletti JP, Kossenkov AV (2011). Targeting ErbB3-mediated stromal-epithelial interactions in pancreatic ductal adenocarcinoma. Br J Cancer.

[78] Jiang N, Wang D, Hu Z (2014). Combination of anti-HER3 antibody MM-121/SAR256212 and cetuximab inhibits tumor growth in preclinical models of head and neck squamous cell carcinoma. Mol Cancer Ther.

[79] Liu JF, Ray-Coquard I, Selle F (2016). Randomized phase II trial of seribantumab in combination with paclitaxel in patients with advanced platinum-resistant or -refractory ovarian cancer. J Clin Oncol.

[80] Kawakami H, Yonesaka K (2016). HER3 and its ligand, heregulin, as targets for cancer therapy. Recent Pat Anticancer Drug Discov.

[81] Meulendijks D, Jacob W, Voest EE (2017). Phase Ib study of lumretuzumab plus cetuximab or erlotinib in solid tumor patients and evaluation of HER3 and heregulin as potential biomarkers of clinical activity. Clin Cancer Res.

[82] Schneeweiss A, Park-Simon TW, Albanell J (2018). Phase Ib study evaluating safety and clinical activity of the anti-HER3 antibody lumretuzumab combined with the anti-HER2 antibody pertuzumab and paclitaxel in HER3-positive, HER2-low metastatic breast cancer. Invest New Drugs.

[83] Meulendijks D, Jacob W, Martinez-Garcia M (2016). First-in-human phase i study of lumretuzumab, a glycoengineered humanized anti-HER3 monoclonal antibody, in patients with metastatic or advanced HER3-positive solid tumors. Clin Cancer Res.

[84] Brinkmann U, Kontermann RE (2021). Bispecific antibodies. Science.

[85] Labrijn AF, Janmaat ML, Reichert JM, Parren PWHI (2019). Bispecific antibodies: a mechanistic review of the pipeline. Nat Rev Drug Discov.

[86] Schram AM, Odintsov I, Espinosa-Cotton M (2022). Zenocutuzumab, a HER2xHER3 bispecific antibody, is effective therapy for tumors driven by NRG1 gene rearrangements. Cancer Discov.

[87] Alsina M, Boni V, Schellens JH (2017). First-in-human phase 1/2 study of MCLA-128, a full length IgG1 bispecific antibody targeting HER2 and HER3: final phase 1 data and preliminary activity in HER2+ metastatic breast cancer (MBC). J Clin Oncol.

[88] Denlinger CS, Alsina Maqueda M, Watkins DJ (2016). Randomized phase 2 study of paclitaxel (PTX), trastuzumab (T) with or without MM-111 in HER2 expressing gastroesophageal cancers (GEC). J Clin Oncol.

[89] Kundranda M, Gracian AC, Zafar SF (2020). Randomized, double-blind, placebo-controlled phase II study of istiratumab (MM-141) plus nab-paclitaxel and gemcitabine versus nab-paclitaxel and gemcitabine in front-line metastatic pancreatic cancer (CARRIE). Ann Oncol.

[90] Lieu CH, Hidalgo M, Berlin JD (2017). A phase Ib dose-escalation study of the safety, tolerability, and pharmacokinetics of cobimetinib and duligotuzumab in patients with previously treated locally advanced or metastatic cancers with mutant KRAS. Oncologist.

[91] Juric D, Dienstmann R, Cervantes A (2015). Safety and pharmacokinetics/pharmacodynamics of the first-in-class dual action HER3/EGFR antibody MEHD7945A in locally advanced or metastatic epithelial tumors. Clin Cancer Res.

[92] Xue J, Kong D, Yao Y (2020). Prediction of human pharmacokinetics and clinical effective dose of SI-B001, an EGFR/HER3 Bi-specific monoclonal antibody. J Pharm Sci.

[93] Gu J, Yang J, Chang Q, Liu Z, Ghayur T, Gu J (2015). Identification of anti-EGFR and anti-ErbB3 dual variable domains immunoglobulin (DVD-Ig) proteins with unique activities. PLoS One.

[94] Rau A, Lieb WS, Seifert O (2020). Inhibition of tumor cell growth and cancer stem cell expansion by a bispecific antibody targeting EGFR and HER3. Mol Cancer Ther.

[95] Rau A, Kocher K, Rommel M (2021). A bivalent, bispecific Dab-Fc antibody molecule for dual targeting of HER2 and HER3. MAbs.

[96] Hassani D, Amiri MM, Mohammadi M (2021). A novel tumor inhibitory hybridoma monoclonal antibody with dual specificity for HER3 and HER2. Curr Res Transl Med.

[97] de Vries Schultink AHM, Doornbos RP, Bakker ABH (2018). Translational PK-PD modeling analysis of MCLA-128, a HER2/HER3 bispecific monoclonal antibody, to predict clinical efficacious exposure and dose. Invest New Drugs.

[98] McDonagh CF, Huhalov A, Harms BD (2012). Antitumor activity of a novel bispecific antibody that targets the ErbB2/ErbB3 oncogenic unit and inhibits heregulin-induced activation of ErbB3. Mol Cancer Ther.

[99] Richards DA, Braiteh FS, Garcia AA (2014). A phase 1 study of MM-111, a bispecific HER2/HER3 antibody fusion protein, combined with multiple treatment regimens in patients with advanced HER2-positive solid tumors. J Clin Oncol.

[100] Yu S, Liu Q, Han X (2017). Development and clinical application of anti-HER2 monoclonal and bispecific antibodies for cancer treatment. Exp Hematol Oncol.

[101] Fitzgerald JB, Johnson BW, Baum J (2014). MM-141, an IGF-IR- and ErbB3-directed bispecific antibody, overcomes network adaptations that limit activity of IGF-IR inhibitors. Mol Cancer Ther.

[102] Camblin AJ, Pace EA, Adams S (2018). Dual inhibition of IGF-1R and ErbB3 enhances the activity of gemcitabine and nab-paclitaxel in preclinical models of pancreatic cancer. Clin Cancer Res.

[103] Schaefer G, Haber L, Crocker LM (2011). A two-in-one antibody against HER3 and EGFR has superior inhibitory activity compared with monospecific antibodies. Cancer Cell.

[104] Jimeno A, Machiels JP, Wirth L (2016). Phase Ib study of duligotuzumab (MEHD7945A) plus cisplatin/5-fluorouracil or carboplatin/paclitaxel for first-line treatment of recurrent/metastatic squamous cell carcinoma of the head and neck. Cancer.

[105] Huang S, Li C, Armstrong EA (2013). Dual targeting of EGFR and HER3 with MEHD7945A overcomes acquired resistance to EGFR inhibitors and radiation. Cancer Res.

[106] De Pauw I, Wouters A, Van den Bossche J (2017). Dual targeting of epidermal growth factor receptor and HER3 by MEHD7945A as monotherapy or in combination with cisplatin partially overcomes cetuximab resistance in head and neck squamous cell carcinoma cell lines. Cancer Biother Radiopharm.

[107] Laterza MM, Ciaramella V, Facchini BA (2021). Enhanced antitumor effect of trastuzumab and duligotuzumab or ipatasertib combination in HER-2 positive gastric cancer cells. Cancers.

[108] Hill AG, Findlay MP, Burge ME (2018). Phase II study of the dual EGFR/HER3 inhibitor duligotuzumab (MEHD7945A) versus cetuximab in combination with FOLFIRI in second-line RAS wild-type metastatic colorectal cancer. Clin Cancer Res.

[109] Marks JA, Wilgucki M, Liu SV, Reuss JE (2022). Antibody-drug conjugates in non-small cell lung cancer: emergence of a novel therapeutic class. Curr Oncol Rep.

[110] Hashimoto Y, Koyama K, Kamai Y (2019). A novel HER3-targeting antibody-drug conjugate, U3-1402, exhibits potent therapeutic efficacy through the delivery of cytotoxic payload by efficient internalization. Clin Cancer Res.

[111] Fu Z, Li S, Han S, Shi C, Zhang Y (2022). Antibody drug conjugate: the “biological missile” for targeted cancer therapy. Signal Transduct Target Ther.

[112] Chau CH, Steeg PS, Figg WD (2019). Antibody-drug conjugates for cancer. Lancet.

[113] Wymant JM, Sayers EJ, Muir D, Jones AT (2020). Strategic trastuzumab mediated crosslinking driving concomitant HER2 and HER3 endocytosis and degradation in breast cancer. J Cancer.

[114] Janne PA, Yu HA, Johnson ML (2019). Safety and preliminary antitumor activity of U3-1402: a HER3-targeted antibody drug conjugate in EGFR TKI-resistant, EGFRm NSCLC. J Clin Oncol.

[115] Capone E, Giansanti F, Ponziani S (2017). EV20-Sap, a novel anti-HER-3 antibody-drug conjugate, displays promising antitumor activity in melanoma. Oncotarget.

[116] Capone E, Tryggvason T, Cela I (2023). HER-3 surface expression increases in advanced colorectal cancer representing a potential therapeutic target. Cell Death Discov.

[117] D’Agostino D, Gentile R, Ponziani S (2021). EV20-sss-vc/MMAF, an HER-3 targeting antibody-drug conjugate displays antitumor activity in liver cancer. Oncol Rep.

[118] Capone E, Lattanzio R, Gasparri F (2021). EV20/NMS-P945, a novel thienoindole based antibody-drug conjugate targeting HER-3 for solid tumors. Pharmaceutics.

[119] Bourillon L, Bourgier C, Gaborit N (2019). An auristatin-based antibody-drug conjugate targeting HER3 enhances the radiation response in pancreatic cancer. Int J Cancer.

[120] Weng W, Meng T, Pu J (2023). AMT-562, a novel HER3-targeting antibody-drug conjugate, demonstrates a potential to broaden therapeutic opportunities for HER3-expressing tumors. Mol Cancer Ther.

[121] Yonesaka K, Takegawa N, Watanabe S (2019). An HER3-targeting antibody-drug conjugate incorporating a DNA topoisomerase I inhibitor U3-1402 conquers EGFR tyrosine kinase inhibitor-resistant NSCLC. Oncogene.

[122] Koganemaru S, Kuboki Y, Koga Y (2019). U3-1402, a novel HER3-targeting antibody-drug conjugate, for the treatment of colorectal cancer. Mol Cancer Ther.

[123] Haikala HM, Lopez T, Köhler J (2022). EGFR inhibition enhances the cellular uptake and antitumor-activity of the HER3 antibody-drug conjugate HER3-DXd. Cancer Res.

[124] Yonesaka K, Tanizaki J, Maenishi O (2022). HER3 augmentation via blockade of EGFR/AKT signaling enhances anticancer activity of HER3-targeting patritumab deruxtecan in EGFR-mutated non-small cell lung cancer. Clin Cancer Res.

[125] Haratani K, Yonesaka K, Takamura S (2020). U3-1402 sensitizes HER3-expressing tumors to PD-1 blockade by immune activation. J Clin Invest.

[126] Jänne PA, Baik C, Su WC (2022). Efficacy and safety of patritumab deruxtecan (HER3-DXd) in EGFR inhibitor-resistant, EGFR-mutated non-small cell lung cancer. Cancer Discov.

[127] Yu HA, Goto Y, Hayashi H (2023). HERTHENA-Lung01, a phase II trial of patritumab deruxtecan (HER3-DXd) in epidermal growth factor receptor-mutated non-small-cell lung cancer after epidermal growth factor receptor tyrosine kinase inhibitor therapy and platinum-based chemotherapy. J Clin Oncol.

[128] Masuda N, Yonemori K, Takahashi S (2019). Abstract PD1-03: single agent activity of U3-1402, a HER3-targeting antibody-drug conjugate, in HER3-overexpressing metastatic breast cancer: updated results of a phase 1/2 trial. Cancer Res.

[129] Capone E, Lamolinara A, D’Agostino D (2018). EV20-mediated delivery of cytotoxic auristatin MMAF exhibits potent therapeutic efficacy in cutaneous melanoma. J Control Release.

[130] Gandullo-Sánchez L, Capone E, Ocaña A, Iacobelli S, Sala G, Pandiella A (2020). HER3 targeting with an antibody-drug conjugate bypasses resistance to anti-HER2 therapies. EMBO Mol Med.

[131] Wang M, Hu Y, Yu T, Ma X, Wei X, Wei Y (2018). Pan-HER-targeted approach for cancer therapy: mechanisms, recent advances and clinical prospect. Cancer Lett.

[132] Jacobsen HJ, Poulsen TT, Dahlman A (2015). Pan-HER, an antibody mixture simultaneously targeting EGFR, HER2, and HER3, effectively overcomes tumor heterogeneity and plasticity. Clin Cancer Res.

[133] Sampera A, Sánchez-Martín FJ, Arpí O (2019). HER-family ligands promote acquired resistance to trastuzumab in gastric cancer. Mol Cancer Ther.

[134] Hickinson DM, Klinowska T, Speake G (2010). AZD8931, an equipotent, reversible inhibitor of signaling by epidermal growth factor receptor, ERBB2 (HER2), and ERBB3: a unique agent for simultaneous ERBB receptor blockade in cancer. Clin Cancer Res.

[135] Mu Z, Klinowska T, Dong X (2014). AZD8931, an equipotent, reversible inhibitor of signaling by epidermal growth factor receptor (EGFR), HER2, and HER3: preclinical activity in HER2 non-amplified inflammatory breast cancer models. J Exp Clin Cancer Res.

[136] Propper DJ, Gao F, Saunders MP (2023). PANTHER: AZD8931, inhibitor of EGFR, ERBB2 and ERBB3 signalling, combined with FOLFIRI: a Phase I/II study to determine the importance of schedule and activity in colorectal cancer. Br J Cancer.

[137] Thomas A, Virdee PS, Eatock M (2020). Dual Erb B Inhibition in Oesophago-gastric Cancer (DEBIOC): a phase I dose escalating safety study and randomised dose expansion of AZD8931 in combination with oxaliplatin and capecitabine chemotherapy in patients with oesophagogastric adenocarcinoma. Eur J Cancer.

[138] Ellebaek S, Brix S, Grandal M (2016). Pan-HER-An antibody mixture targeting EGFR, HER2 and HER3 abrogates preformed and ligand-induced EGFR homo- and heterodimers. Int J Cancer.

[139] Rabia E, Garambois V, Hubert J (2021). Anti-tumoral activity of the Pan-HER (Sym013) antibody mixture in gemcitabine-resistant pancreatic cancer models. MAbs.

[140] Reddy TP, Choi DS, Anselme AC (2020). Simultaneous targeting of HER family pro-survival signaling with Pan-HER antibody mixture is highly effective in TNBC: a preclinical trial with PDXs. Breast Cancer Res.

[141] Rau A, Janssen N, Kühl L (2022). Triple targeting of HER receptors overcomes heregulin-mediated resistance to EGFR blockade in colorectal cancer. Mol Cancer Ther.

[142] Berlin J, Tolcher AW, Ding C (2022). First-in-human trial exploring safety, antitumor activity, and pharmacokinetics of Sym013, a recombinant pan-HER antibody mixture, in advanced epithelial malignancies. Invest New Drugs.

[143] Osada T, Hartman ZC, Wei J (2018). Polyfunctional anti-human epidermal growth factor receptor 3 (anti-HER3) antibodies induced by HER3 vaccines have multiple mechanisms of antitumor activity against therapy resistant and triple negative breast cancers. Breast Cancer Res.

[144] Osada T, Morse MA, Hobeika A (2017). Vaccination targeting human HER3 alters the phenotype of infiltrating T cells and responses to immune checkpoint inhibition. Oncoimmunology.

[145] Miller MJ, Foy KC, Overholser JP, Nahta R, Kaumaya PT (2014). HER-3 peptide vaccines/mimics: combined therapy with IGF-1R, HER-2, and HER-1 peptides induces synergistic antitumor effects against breast and pancreatic cancer cells. Oncoimmunology.

[146] Kaumaya PT (2020). B-cell epitope peptide cancer vaccines: a new paradigm for combination immunotherapies with novel checkpoint peptide vaccine. Future Oncol.

[147] Lipkowitz S (2003). The role of the ubiquitination-proteasome pathway in breast cancer: ubiquitin mediated degradation of growth factor receptors in the pathogenesis and treatment of cancer. Breast Cancer Res.

[148] Fan Q, Wang Q, Cai R, Yuan H, Xu M (2020). The ubiquitin system: orchestrating cellular signals in non-small-cell lung cancer. Cell Mol Biol Lett.

[149] Pinilla-Macua I, Sorkin A (2023). Cbl and Cbl-b independently regulate EGFR through distinct receptor interaction modes. Mol Biol Cell.

[150] Luan H, Bailey TA, Clubb RJ (2021). CHIP/STUB1 ubiquitin ligase functions as a negative regulator of ErbB2 by promoting its early post-biosynthesis degradation. Cancers.

[151] Feng SM, Muraoka-Cook RS, Hunter D (2009). The E3 ubiquitin ligase WWP1 selectively targets HER4 and its proteolytically derived signaling isoforms for degradation. Mol Cell Biol.

[152] Qiu XB, Goldberg AL (2002). Nrdp1/FLRF is a ubiquitin ligase promoting ubiquitination and degradation of the epidermal growth factor receptor family member, ErbB3. Proc Natl Acad Sci U S A.

[153] Shi H, Gong H, Cao K (2015). Nrdp1-mediated ErbB3 degradation inhibits glioma cell migration and invasion by reducing cytoplasmic localization of p27(Kip1). J Neurooncol.

[154] Yen L, Cao Z, Wu X (2006). Loss of Nrdp1 enhances ErbB2/ErbB3-dependent breast tumor cell growth. Cancer Res.

[155] Huang Z, Choi BK, Mujoo K (2015). The E3 ubiquitin ligase NEDD4 negatively regulates HER3/ErbB3 level and signaling. Oncogene.

[156] Verma N, Müller AK, Kothari C (2017). Targeting of PYK2 synergizes with EGFR antagonists in basal-like TNBC and circumvents HER3-associated resistance via the NEDD4-NDRG1 axis. Cancer Res.

[157] (2021). Cardoso AP, Banerjee M, Nail AN, Lykoudi A, States JC. miRNA dysregulation is an emerging modulator of genomic instability. Semin Cancer Biol.

[158] He B, Zhao Z, Cai Q (2020). miRNA-based biomarkers, therapies, and resistance in Cancer. Int J Biol Sci.

[159] Plantamura I, Cataldo A, Cosentino G, Iorio MV (2020). miR-205 in breast cancer: state of the art. Int J Mol Sci.

[160] Iorio MV, Casalini P, Piovan C (2009). microRNA-205 regulates HER3 in human breast cancer. Cancer Res.

[161] Hao Y, Li J, Zhang H, Guan G, Guo Y (2020). MicroRNA-205 targets HER3 and suppresses the growth, chemosensitivity and metastasis of human nasopharyngeal carcinoma cells. J buon.

[162] Scott GK, Goga A, Bhaumik D, Berger CE, Sullivan CS, Benz CC (2007). Coordinate suppression of ERBB2 and ERBB3 by enforced expression of micro-RNA miR-125a or miR-125b. J Biol Chem.

[163] Lyu H, Huang J, He Z, Liu B (2018). Targeting of HER3 with functional cooperative miRNAs enhances therapeutic activity in HER2-overexpressing breast cancer cells. Biol Proced Online.

[164] Zhao Z, Li R, Sha S, Wang Q, Mao W, Liu T (2014). Targeting HER3 with miR-450b-3p suppresses breast cancer cells proliferation. Cancer Biol Ther.

[165] Casamassimi A, Ciccodicola A, Rienzo M (2023). Transcriptional regulation and its misregulation in human diseases. Int J Mol Sci.

[166] Li Y, Azmi AS, Mohammad RM (2022). Deregulated transcription factors and poor clinical outcomes in cancer patients. Semin Cancer Biol.

[167] Lourenco C, Resetca D, Redel C (2021). MYC protein interactors in gene transcription and cancer. Nat Rev Cancer.

[168] Suen TC, Hung MC (1991). c-myc reverses neu-induced transformed morphology by transcriptional repression. Mol Cell Biol.

[169] Sun C, Hobor S, Bertotti A (2014). Intrinsic resistance to MEK inhibition in KRAS mutant lung and colon cancer through transcriptional induction of ERBB3. Cell Rep.

[170] Calissi G, Lam EW, Link W (2021). Therapeutic strategies targeting FOXO transcription factors. Nat Rev Drug Discov.

[171] Garrett JT, Olivares MG, Rinehart C (2011). Transcriptional and posttranslational up-regulation of HER3 (ErbB3) compensates for inhibition of the HER2 tyrosine kinase. Proc Natl Acad Sci U S A.

[172] Chakrabarty A, Sánchez V, Kuba MG, Rinehart C, Arteaga CL (2012). Feedback upregulation of HER3 (ErbB3) expression and activity attenuates antitumor effect of PI3K inhibitors. Proc Natl Acad Sci U S A.

[173] Abel EV, Aplin AE (2010). FOXD3 is a mutant B-RAF-regulated inhibitor of G(1)-S progression in melanoma cells. Cancer Res.

[174] Han S, Ren Y, He W (2018). ERK-mediated phosphorylation regulates SOX10 sumoylation and targets expression in mutant BRAF melanoma. Nat Commun.

[175] Abel EV, Basile KJ, Kugel CH 3rd (2013). Melanoma adapts to RAF/MEK inhibitors through FOXD3-mediated upregulation of ERBB3. J Clin Invest.

[176] Raap M, Gierendt L, Kreipe HH, Christgen M (2021). Transcription factor AP-2beta in development, differentiation and tumorigenesis. Int J Cancer.

[177] Zhu CH, Domann FE (2002). Dominant negative interference of transcription factor AP-2 causes inhibition of ErbB-3 expression and suppresses malignant cell growth. Breast Cancer Res Treat.

[178] Jacob W, James I, Hasmann M, Weisser M (2018). Clinical development of HER3-targeting monoclonal antibodies: perils and progress. Cancer Treat Rev.

[179] Wu Y, Zhang Y, Wang M (2013). Downregulation of HER3 by a novel antisense oligonucleotide, EZN-3920, improves the antitumor activity of EGFR and HER2 tyrosine kinase inhibitors in animal models. Mol Cancer Ther.

[180] Sarup J, Jin P, Turin L (2008). Human epidermal growth factor receptor (HER-1:HER-3) Fc-mediated heterodimer has broad antiproliferative activity in vitro and in human tumor xenografts. Mol Cancer Ther.

[181] Yonesaka K, Hirotani K, von Pawel J (2017). Circulating heregulin level is associated with the efficacy of patritumab combined with erlotinib in patients with non-small cell lung cancer. Lung Cancer.

[182] Ocaña A, Díez-González L, Esparís-Ogando A, Montero JC, Amir E, Pandiella A (2016). Neuregulin expression in solid tumors: prognostic value and predictive role to anti-HER3 therapies. Oncotarget.

